# An enhanced artificial bee colony algorithm (EABC) for solving dispatching of hydro-thermal system (DHTS) problem

**DOI:** 10.1371/journal.pone.0189282

**Published:** 2018-01-11

**Authors:** Yi Yu, Yonggang Wu, Binqi Hu, Xinglong Liu

**Affiliations:** 1 School of Hydropower and Information Engineering, Huazhong University of Science and Technology, Wuhan, China; 2 Dispatching and Communication Bureau, State Grid Hunan Electric Power Company, Changsha, China; Tianjin University, CHINA

## Abstract

The dispatching of hydro-thermal system is a nonlinear programming problem with multiple constraints and high dimensions and the solution techniques of the model have been a hotspot in research. Based on the advantage of that the artificial bee colony algorithm (ABC) can efficiently solve the high-dimensional problem, an improved artificial bee colony algorithm has been proposed to solve DHTS problem in this paper. The improvements of the proposed algorithm include two aspects. On one hand, local search can be guided in efficiency by the information of the global optimal solution and its gradient in each generation. The global optimal solution improves the search efficiency of the algorithm but loses diversity, while the gradient can weaken the loss of diversity caused by the global optimal solution. On the other hand, inspired by genetic algorithm, the nectar resource which has not been updated in limit generation is transformed to a new one by using selection, crossover and mutation, which can ensure individual diversity and make full use of prior information for improving the global search ability of the algorithm. The two improvements of ABC algorithm are proved to be effective via a classical numeral example at last. Among which the genetic operator for the promotion of the ABC algorithm’s performance is significant. The results are also compared with those of other state-of-the-art algorithms, the enhanced ABC algorithm has general advantages in minimum cost, average cost and maximum cost which shows its usability and effectiveness. The achievements in this paper provide a new method for solving the DHTS problems, and also offer a novel reference for the improvement of mechanism and the application of algorithms.

## Introduction

Research on the dispatching of hydro-thermal system (DHTS) to achieve the balance of supply and demand is an issue which has significant economic benefits [1]. The specific content of DHTS is that the hydro-thermal power stations are controlled to make the combined output to meet the load demand over scheduling period, and all controlled variables should be met the corresponding constraints at the same time. Mathematically, the DHTS problem essentially is a nonlinear, nonconvex programming problem with high-dimensions and multi-constraints. Seeking for effective methods for solving such problems has been a headache and a hot spot over the past decades.

In recent years, researchers have proposed a number of optimization algorithms in succession, which can be divided into two categories on the whole: traditional optimization algorithms and modern intelligent algorithms [2]. The problems solved by traditional optimization algorithms mostly belong to convex optimization problems that have only one definite global optimum. On the contrary, the modern intelligent algorithm is suitable for solving nonconvex optimization problems, especially for multi-extremum problems. A large number of nonconvex optimization problems exist in most fields, such as economic science, industrial production and network optimization [3–5]. The application of modern intelligent algorithms has naturally become the focus of attention in various fields, such as artificial neural network [6], genetic algorithm [7], artificial immune algorithm [8], ant colony algorithm [9], particle swarm algorithm [10], artificial fish swarm algorithm [11], cultural algorithm [12], tabu search algorithm [13] and simulated annealing algorithm etc [14–24]. These algorithms have their own advantages in solving these various problems. Nevertheless, no single intelligent algorithm can take all advantages and anyone has more or less defects such as dimensional difficulties, large memory requirements or inability to handle nonlinear characteristics, premature phenomena and falling into local optima and taking too much computation time [25].

The ABC algorithm which is proposed by Karaboga in 2005 is a novel global optimization algorithm which is based on swarm intelligence [26]. The intuitive background of the ABC algorithm is the behavior of bee colony, which shares and exchanges the colony information for discovering the optimal nectar resource. The standard ABC with simple operation, high precision and strong robustness is good at global searching and almost has no requirements on the objective function and constraints, which basically does not use external information. Compared with other intelligence algorithms, the prominent advantage of ABC is that both global and local search are used in its each iteration, and so that the probability of finding the optimal solution is greatly increased [27]. In view of these, the ABC algorithm has been introduced to solve optimization problem of dispatching hydrothermal system in reference [28]. Considering the ABC algorithm's characteristics of well exploration and poor exploitation, researchers have put forward many effective improvements to this algorithm [29–38]. GABC is one of these improved algorithms, which can improve the exploitation ability of the algorithm by using the global optimal solution [39]. However, research [27] points out that the improved measure may reduce the algorithm's global optimization ability to some extent. In order to optimize the performance of the algorithm further, this paper improves it from two aspects. On one hand, the gradient information of the global optimal solution is introduced into the search formula for properly weakening the global optimal solution's guiding effect, which results in a fully search in the neighborhood under a lower convergence speed. On the other hand, inspired by the genetic algorithm, the mechanism of crossover and mutation is introduced in the scouters' global search, in which crossover can make full use of prior information and mutation can ensure the diversity of individuals, which make the random searchers obtain extra nectar information thus improve their search efficiency. Prior to this, some mixed algorithms about ABC and GA have been proposed in reference [27], [40] and [41] et.al. In these references, their genetic operators are all implemented to manipulate all the populations in each generation so as to prefer superior species to the next generation. These may lead to unsatisfactory optimization results due to incomplete local search and premature optimization. This paper tried to use genetic operator to generate new nectar resource in the case of that any nectar resource has not been updated exceeding the limit, which can effectively avoid the premature convergence, and make full use of the prior information and ensure the diversity of the population. Besides, three pretreatment measures that contain the adaptive flow decomposition strategy, the penalty function, the selection probability, are performed to improve the solving efficiency of the algorithm in this paper. These measures can better achieve the balance between the exploration and exploitation ability of the ABC algorithm in theory and a classical test system was used to attest the improved algorithm's rationality and superiority in this paper at last.

## Mathematical model

### Economic dispatch model of DHTS

Economic dispatch of hydro-thermal system is an important branch of DHTS. Economic dispatch of hydro-thermal system minimizes the total cost of hydro-thermal power under the premise of ensuring DHTS to balance the power load demand at each time interval. The generation principle of hydro plant with reservoir is to redistribute the natural upstream river for achieving artificial control of power generation by regulating its reservoir storage. In general, the daily economic dispatch of hydro-thermal problem is aimed to minimize the total thermal cost and the generation cost of thermal unit can be regarded as a quadratic function of thermal unit's output [25, 42–44]. Therefore, the daily economic dispatch of hydro-thermal problem can be modelled as follow,

$$obj:\;\min\overset{T}{\underset{t=1}{\sum }}\overset{N_{s}}{\underset{i=1}{\sum }}F_{i}(P_{s}(i,t))=\min\overset{T}{\underset{t=1}{\sum }}\overset{N_{s}}{\underset{i=1}{\sum }}\left[a_{i}+b_{i}∙P_{s}(i,t)+c_{i}∙{P_{s}(i,t)}^{2}\right]$$ (1)

Where *P*_*s*_(*i*,*t*) is power generation of the *i*th thermal unit at time interval t. *F*_*i*_ is cost function of the *i*th thermal unit. *N*_*s*_ is number of thermal units. *T* is number of time intervals. *a*_*i*_, *b*_*i*_ and c_*i*_ are the cost coefficients of the *i*th thermal unit, and their values are related to the performance of thermal unit.

### Boundary conditions

It is necessary to consider all restrictions over scheduling period, it includes the power balance limits, the hydropower system limits and the thermal power system limits etc. Specific performances are shown as follows,

#### (a) The power balance limits

$$\overset{N_{h}}{\underset{i=0}{\sum }}P_{h}(i,t)+\overset{N_{s}}{\underset{j=0}{\sum }}P_{s}(i,t)=P_{t}\;(t=1,2\cdots T)$$ (2)

Where *P*_*h*_(*i*,*t*) is the power generation of the *i*th hydro plant at time interval *t*. *N*_*h*_ is the amount of hydro plants. *P*_*t*_ is the system load demand at time interval *t*.

#### (b) The generation limits of each thermal unit

$$P_{si}^{min}\leq P_{s}(i,t)\leq P_{si}^{max}\;(i=1,2\cdots N_{s};t=1,2\cdots T)$$ (3)

Where *P*_*si*_^*min*^ and *P*_*si*_^*max*^ are the minimum and maximum generation of *i*th thermal unit, respectively.

#### (c) The generation limits of hydro plant

$$P_{hi}^{min}\leq P_{h}(i,t)\leq P_{hi}^{max}\;(i=1,2\cdots N_{h};t=1,2\cdots T)$$ (4)

Where *P*_*hi*_^*min*^ and *P*_*hi*_^*max*^ are the minimum and maximum generation of *i*th hydro plant, respectively.

#### (d) Reservoir capacity constraints

$$\left\{ \begin{array}{l} V_{i}^{min}\leq V(i,t)\leq V_{i}^{max}\\ V(i,0)=V_{i}^{initial}\\ V(i,T)=V_{i}^{end} \end{array}\;\left(i=1,2\cdots N_{h};t=1,2\cdots T\right)\right.$$ (5)

Where *V*_*i*_^*min*^ and *V*_*i*_^*max*^ are the minimum and maximum storage capacity of *i*th hydro plant, respectively. *V*(*i*,*t*) is the storage capacity of the *i*th hydro plant at the end of time interval *t*. *V*_*i*_^*initial*^ and *V*_*i*_^*end*^ are the initial and end storage capacity of *i*th hydro plant, respectively.

#### (e) Discharge restrictions

$$Q_{i}^{min}\leq Q(i,t)\leq Q_{i}^{max}\;(i=1,2\cdots N_{h};t=1,2\cdots T)$$ (6)

Where *Q*_*i*_^*min*^ and *Q*_*i*_^*max*^ are the minimum and maximum discharge of *i*th hydro plant, respectively. *Q*(*i*,*t*) is the discharge of *i*th hydro plant at time interval *t*.

#### (f) The continuity equation

$$V(i,t+1)=V(i,t)+q(i,t)+\overset{N_{i}}{\underset{j=1}{\sum }}(Q_{j}^{t-\tau_{j}}+S_{j}^{t-\tau_{j}})-Q(i,t)-S_{i}^{t}\;(i=1,2\cdots N_{h};t=1,2\cdots T)$$ (7)

Where *q*(*i*,*t*) is the interval inflow of *i*th hydro plant at time interval *t*. *N*_*i*_ is the number of direct upstream hydro plants. *τ*_*j*_ is the transport delay time of *j*th direct upstream hydro plant. *S*_*i*_^*t*^ is the spillage of *i*th hydro plant at time interval *t*.

#### (g) Hydraulic generation characteristics

The power generation of hydro plant is directly related to water head and discharge. There is a correlation between water head and reservoir capacity, which is affected by the shape of the reservoir, it can be expressed as follow,

$$\left\{ \begin{array}{l} P_{h}(i,t)=f_{1}(Q(i,t),H(i,t))\\ V(i,t)=f_{2}(H(i,t)) \end{array}\right.\;(i=1,2\cdots N_{h};t=1,2\cdots T)$$ (8)

Therefore, hydropower generation can be seen as a function of discharge and storage capacity in a certain time.Studies [25, 42–44] have pointed out that it can be formulated as follows,

$$P_{h}(i,t)=C_{1}∙Q{\left(i,t\right)}^{2}+C_{2}∙V{\left(i,t\right)}^{2}+C_{3}∙Q(i,t)∙V(i,t)+C_{4}∙Q(i,t)+C_{5}∙V(i,t)+C_{6}$$ (9)

Where *C*_1_, *C*_2_, *C*_3_, *C*_4_, *C*_5_ and *C*_6_ are the performance parameters of hydro plant, their values are related to the generation performance.

## Overview of artificial bee colony algorithm

### The principle of ABC algorithm

The standard ABC algorithm is one of swarm intelligence optimization algorithms, which simulates the bees’ behavior of gathering honey. In ABC algorithm, the bees are divided into three categories based on their division of labor: employees, onlookers and scouters. Employees seek for the nectars, and share the nectars’ information with onlookers in dance area, onlookers select their own nectar based on the shared information and scouters’ duties are to randomly search for nectars. During the process of searching in the standard ABC algorithm, employees and onlookers are responsible for exploration, while scouters are responsible for exploitation.The process of ABC algorithm for solving problem actually is the process of searching in potential solution space. The position of each nectar resource represents a feasible solution to the problem and the amount of nectar indicates the corresponding solution's fitness. In search of each generation, the number of employees is equal to the number of nectar resources, and there is a one-to-one relationship between the employees and nectar resources.

In order to ensure the diversity of population, employees are required to carry on a local search for better nectar resources around the corresponding resources in each generation based on the following formula,

$$\bar{x_{ij}}=x_{ij}+rand(-1,1)∙(x_{ij}-x_{kj})$$ (10)

Where $\bar{xij}$ is the value of generated nectar resource in *j*th dimension, *x*_*ij*_ is the value of *i*th nectar resource in *j*th dimension, *x*_*kj*_ is the value of *k*th nectar resource in *j*th dimension, in which *k* is a random number that is less than population quantity and not equal to *i*. Comparing the generated nectar resource with the original one, the one of higher fitness value is retained by using the greedy selection strategy. The fitness value of nectar resource usually associates with the objective function value, and the calculation is as follow,

$${Fit}_{i}=\left\{ \begin{array}{lll} \frac{1}{1+f_{i}} & ,if & f_{i}>0\\ 1+|f_{i}| & ,if & f_{i}\leq 0 \end{array}\right.$$ (11)

According to the information of nectar resources transmitted by employees, each onlooker will choose a nectar resource based on roulette strategy. The formula of possibility of being selected is as follow,

$$p_{i}=\frac{{Fit}_{i}}{\sum_{j=1}^{n}{Fit}_{j}}$$ (12)

Where *Fit*_*i*_ is the fitness value of *i*th nectar resource and *n* is the number of nectar resources. The onlookers search for new nectar resources according to the Eq (11) after selecting nectar resources by roulette strategy. Meanwhile, employees update the nectar resources by fitness value on the basis of the greedy selection strategy. If any nectar resource has not been updated within a given limit of generation, the corresponding employee gives up the nectar resource, changes the role to be a scouter and searches for a new nectar resource randomly. The scouters search for new nectar resources according to the following formula,

$$x_{id}=x_{id}^{min}+rand(0,1)∙(x_{id}^{max}-x_{id}^{min})$$ (13)

Where *x*_*id*_ is the value of *i*th nectar resource in *d*th dimension. *x*_*id*_^*min*^ and *x*_*id*_^*max*^ are the lower and upper bounds of *i*th nectar resource in *d*th dimension, respectively.

### The specific steps of artificial bee colony algorithm

#### Initialise

#### Repeat

- Keep one-to-one relationship between employes and nectar resources and update information of nectar resources by Eq (10) and determine the fitness value of nectar resources by Eq (11)
- The onlookers select nectar resources according to the information provided by employes based on roulette strategy through Eq (12), search for a nectar resource according to Eq (10) and determine the fitness value of nectar resources
- Determine the scouters, and look for new nectar resources by Eq (13)
- Record the best nectar resource by far

#### Determine whether the terminal condition is satisfied

## Introduction of an enhanced ABC algorithm

The exploitation and exploration are two important aspects of the performances of swarm intelligent algorithms [45, 46]. The former is reflected in algorithms’ local search capability and the latter is reflected in the global search capability. The standard ABC algorithm’s local search is performed by employees and onlookers in each generation and global searching is mainly reflected in the search process of scouters. These two abilities of the standard ABC algorithm will be analyzed and improved in following passage and an enhanced ABC algorithm (*hereafter termed EABC*) is proposed from these improvements in this paper.

### Local search by using gradient information of the global optimal solution

Local search is based on Eq (10) in standard artificial bee colony algorithm. It will be found by analyzing the Eq (10) that the standard ABC algorithm’s local search is to select one dimension from one nectar resource as its local optimization variable, take the selected dimension of this nectar resource as the center and regard projection distance between the nectar resource and another one in this dimension as the search scope. In Eq (14), the coefficient is a totally random number in [–1, 1], *x*_*k*,*j*_ is a random individual in the population and the possibility of selecting a good solution is nearly equal to that of selecting a bad solution. Therefore, the new candidate solution is not promising to be a solution better than the previous one and it also confirms the statement that the artificial bee colony algorithm is tired of local search. To solve this problem, the literature[39] presents GABC to introduce the global optima into the search formula of artificial bee colony algorithm for improving the exploitation which refers to particle swarm optimization and the specific is shown in Eq (14). The validity of this result has been confirmed in reference [39].

$$\bar{x_{ij}}=x_{ij}+\varphi_{ij}∙(x_{ij}-x_{kj})+\beta ∙(x_{j}^{Global}-x_{ij})$$ (14)

**Fig 1:**
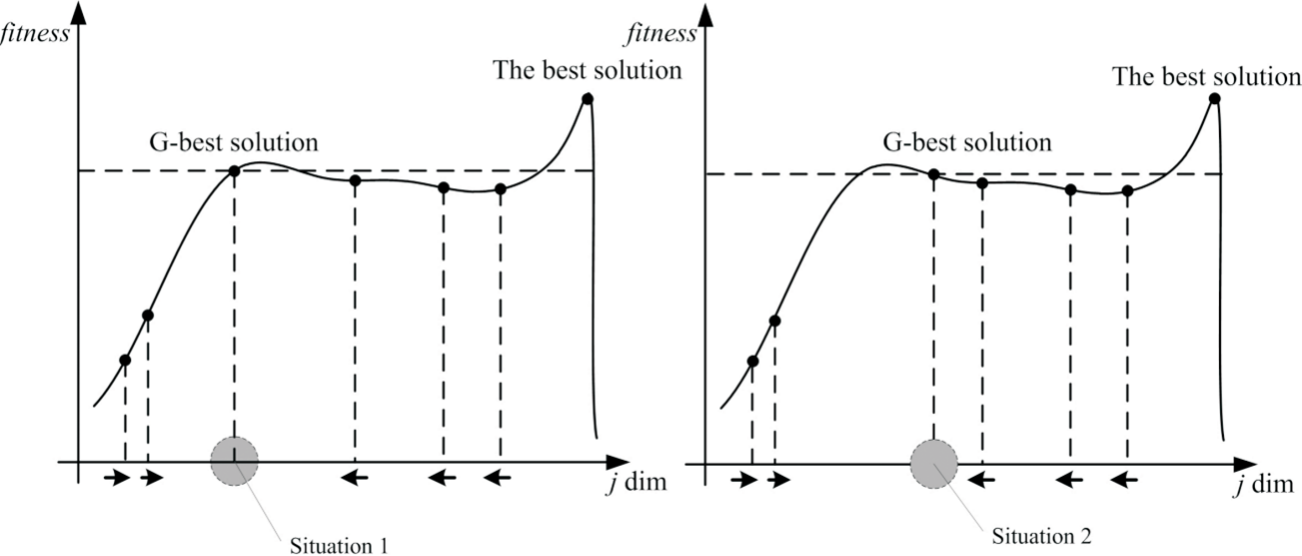
Situations in advanced steps in GABC.

But studies [27] have shown that convergence of the algorithm is too quick to reduce the algorithm's global search ability in a certain extent (Fig 1). In order to weaken the influence on the algorithm's global search by the third of Eq (14), the gradient of G-best solution is introduced in this paper. The G-best solution is marked as *x*^*global*^, which is expressed as (*x*_1_,*x*_2_, …,*x*_D_). The G-best solution can be (*x*_1_+Δ*x*, *x*_2_, …,*x*_D_), (*x*_1_,*x*_2_+Δ*x*, …,*x*_D_), …, (*x*_1_,*x*_2_, …, *x*_D-1_+Δ*x*, *x*_D_) or (*x*_1_,*x*_2_, …, *x*_D-1_, *x*_D_+Δ*x*) while Δ*x* is added in its each dimension respectively. The gradient of G-best solution can be expressed as follows,

$$\left\{ \begin{array}{c} \frac{\partial f}{\partial x_{1}}=\underset{\Delta x\to 0}{\lim}\frac{f(x_{1}+\Delta x,x_{2},\cdots x_{D})-f(x_{1},x_{2},\cdots x_{D})}{\Delta x}\\ \frac{\partial f}{\partial x_{2}}=\underset{\Delta x\to 0}{\lim}\frac{f(x_{1},x_{2}+\Delta x,\cdots x_{D})-f(x_{1},x_{2},\cdots x_{D})}{\Delta x}\\ \vdots \\ \frac{\partial f}{\partial x_{D}}=\underset{\Delta x\to 0}{\lim}\frac{f(x_{1},x_{2},\cdots x_{D}+\Delta x)-f(x_{1},x_{2},\cdots x_{D})}{\Delta x} \end{array}\right.$$ (15)

$${grad}^{Global}=(\frac{\partial f}{\partial x_{1}},\frac{\partial f}{\partial x_{2}}\cdots \frac{\partial f}{\partial x_{D}})$$ (16)

$$|{grad}^{Global}|=\sqrt{{\left(\frac{\partial f}{\partial x_{1}}\right)}^{2}+{\left(\frac{\partial f}{\partial x_{2}}\right)}^{2}+\cdots {\left(\frac{\partial f}{\partial x_{D}}\right)}^{2}}$$ (17)

$${grad}_{e}^{Gloabal}=\frac{\left(\frac{\partial f}{\partial x_{1}},\frac{\partial f}{\partial x_{2}}\cdots \frac{\partial f}{\partial x_{D}}\right)}{\sqrt{{\left(\frac{\partial f}{\partial x_{1}}\right)}^{2}+{\left(\frac{\partial f}{\partial x_{2}}\right)}^{2}+\cdots {\left(\frac{\partial f}{\partial x_{D}}\right)}^{2}}}$$ (18)

Where *f* is analog fitness function and *f* (*x*_1_,*x*_2_, …,*x*_D_) expresses the fitness of nectar resource *x*^*global*^. Δ*x* is a small step size. *grad*^*Global*^ is the gradient of G-best solution.| *grad*^*Global*^| is the gradient norm of global optimal solution. *grad*_*e*_^*Global*^ is the unit gradient vector of global optimal solution.

In this paper, the convergence rate is reduced by fine adjusting the advanced steps in different situations according to gradient direction of the G-best solution. The adjustment is related to the G-best solution’s gradient and the distance between the feasible solution and G-best solution. Thus the search formula can be expressed as follows,

$$x_{ij}^{\prime }=x_{ij}+\varphi_{ij}∙(x_{ij}-x_{kj})+\beta ∙(x_{j}^{Global}-x_{ij})+{\left(-1\right)}^{k}∙\gamma (t)∙|x_{j}^{Global}-x_{ij}|∙|{grad}_{e,j}^{Global}|$$ (19)

$$k=\left\{ \begin{array}{l} even\;,if\;x_{ij}>x_{j}^{Global}\\ odd\;,if\;x_{ij}<x_{j}^{Global} \end{array}\right.$$ (20)

Here, a normal distribution is selected to represent the adaptive coefficients,

$$\gamma (t)=\frac{N}{\sqrt{2\pi }}∙e^{\left[-\frac{t^{2}}{2∙{\left(\frac{N}{4}\right)}^{2}}\right]}$$ (21)

Where *k* is direction control parameter, *x*_*j*_^*global*^ is the value of *j*th dimension of global optimal solution. *γ*(*t*) is adaptive coefficients, *grad*_*e*,*j*_^*Global*^ is the value of *j*th dimension of unit gradient of global optimal solution.

### The idea of selection, crossover and mutation for global search

The set *limit* plays a critical role in global searching capacity of ABC algorithm [27]. When the accumulative generation that nectar resource has not been updated comes to limit, the corresponding employee converts its role to be a scouter and randomly selects a new nectar resource in the solution space instead of the original one. This mechanism is a characteristic of ABC algorithm, which enables the original employee that fall into local optimum to jump out of the local convergence.

As is known, genetic algorithm has the characteristics of self-organization, self-adaptation and self-learning, which is based on the idea of "survival of the fittest"[47]. Genetic algorithm achieves the evolution of populations through the selection, crossover and mutation. Among of them, the selection operator is to contain the optimized individual to the next generation directly or select the parent individuals to prepare for crossover or mutation, the crossover operator generates two new individuals by recombination of two parent individuals, and the mutation operator changes value of a gene from a group of individuals. The genetic algorithm can balance the ability between global and local search through crossover cooperating with mutation.

In the standard ABC algorithm, the employee performs a global search which randomly selects a nectar resource after becoming a scouter and searches by Eq (13). However, searching in a complex solution space randomly is undoubtedly lack of efficiency, especially more obvious in the later optimization. Therefore, this paper supposes that the global search efficiency of ABC algorithm can be enhanced if it can make full use of the prior information of nectar resources.

Inspired by the genetic algorithm, the nectar resource that is generated by the way of selection, crossover and mutation substitutes the original one while the employee is transformed to be a scouter who seeks for a new nectar resource, which will guide the scouter's global search. There is a large probability of crossover and a small fraction probability of the variance. The crossover operation is to select two parent chromosomes based on the selection probability and then generate a new chromosome in coding mechanism of real number. While the mutation operation persists the original operation of random selection of standard ABC algorithm. The search formula is formulated as follow.

$$x_{id}=\left\{ \begin{array}{l} \lambda ∙x_{kd}+(1-\lambda )∙x_{ld}\;,if\;0\leq p\leq p_{c}\\ x_{id}^{min}+rand(0,1)∙(x_{id}^{max}-x_{id}^{min})\;,if\;p_{c}<p\leq 1 \end{array}\right.$$ (22)

Where *λ* is random number and *p*_*c*_ is crossover probability. *K* and *l* are selected based on roulette strategy through Eq (12).

## EABC for solving DHTS problems

The DHTS problems aim to find an economic dispatch scheme to guide the operation of the hydro-thermal system. The proposed EABC algorithm is used to solve DHTS problems and three strategies have been performed.

### Three strategys for EABC solving DHTS problems

#### Strategy 1: Initial flow processing

Since initial storage capacity and end storage capacity of reservoirs are known condition, the front 23 discharges are initialized within the discharge limits and then the final discharge (*Q*_24_) is calculated by Eq (7) so as to meet the water balance of reservoirs. The results of experiments show that the calculated discharge is beyond the discharge limit in a great probability. The probability of initialized effective nectar resources will be very low if the over-limit discharge has not been treated, which directly affects the convergence rate of the algorithm. On the other hand, heavy-treated will reduce the diversity of population. Therefore, this paper proposes a pretreatment measure of the initialized discharge balance between the diversity and convergence rate.

The average value *Q*__avg_ is respectively compared with maximum discharge *Q*_*max*_ and minimum discharge *Q*_*min*_. There are three kinds of potential situations: *Q*_*min*_≤*Q*__avg_≤*Q*_*max*_, *Q*__avg_>*Q*_*max*_ and *Q*__avg_<*Q*_*min*_. The average value *Q*__avg_ is calculated by Eq (23),

Qavg=∑i=024Qi24 (23)

- Under the situation of *Q*_*min*_≤*Q*__avg_≤*Q*_*max*_

The exceeding limit size of *Q*_24_ is measured byΔ*Q*_1_, and then Δ*Q*_1_ is distributed successively to forward period discharge. The front 23 discharges, marked $\bar{Q_{1}}$,…, $\bar{Q_{23}}$, will receive the distributions. In order to reduce the effect of pretreatment on the diversity of population, the order of these 23 discharges is a random sequence of the original discharges, and its motion is shown as following formula (24).

$$\left\{ \begin{array}{l} \Delta Q_{1}=Q_{24}-Q_{max}\\ {\left(\bar{Q_{1}}\cdots \bar{Q_{23}}\right)}_{sequence}=r{and(Q_{1}\cdots Q_{23})}_{sequence} \end{array}\right.$$ (24)

There are below two situations after the first distribution:

$$\left\{ \begin{array}{l} \bar{Q_{23}}=\Delta Q_{1}+\bar{Q_{23}}\;and\;\Delta Q_{2}=0\;,if\;\Delta Q_{1}+\bar{Q_{23}}\leq Q_{max}\\ \bar{Q_{23}}=Q_{max}\;and\;\Delta Q_{2}=\Delta Q_{1}+\bar{Q_{23}}-Q_{max}\;,if\;\Delta Q_{1}+\bar{Q_{23}}>Q_{max} \end{array}\right.$$ (25)

**Fig 2:**
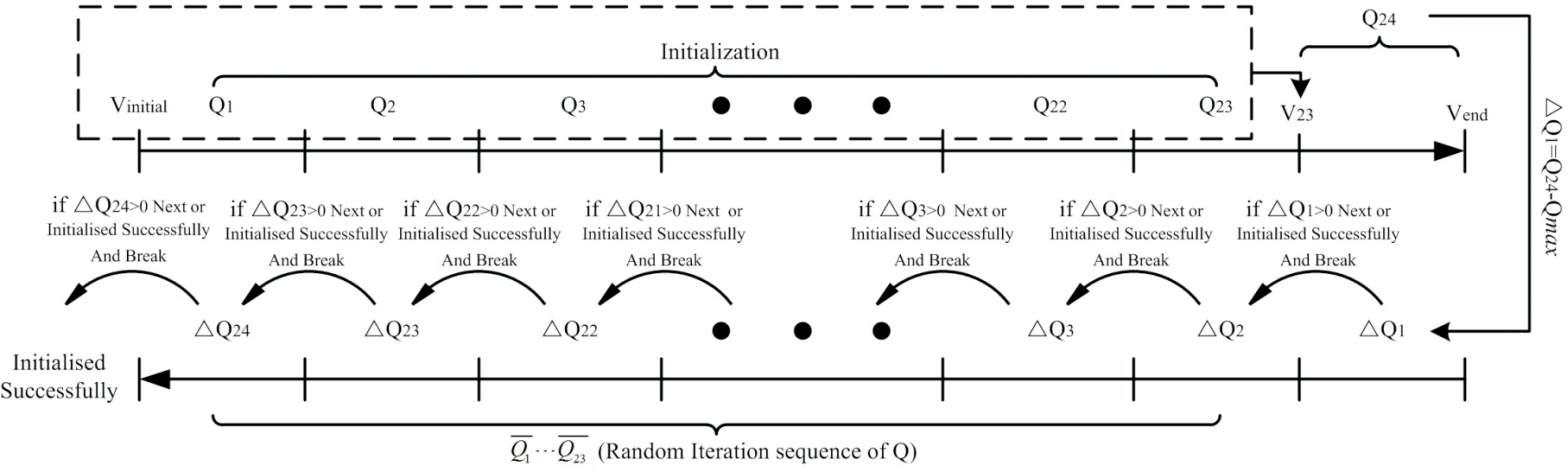
The detailed steps of initial flow processing.

If Δ*Q*_2_ = 0, distribution is over, or Δ*Q*_2_ is distributed repeatly to forward period of discharge until the assigned discharge is reduced to zero. The detailed steps are shown in Fig 2, then the modified nectar resourrce can meet the discharge restrictions.

- Under the situation of *Q*__avg_>*Q*_*max*_

There is still residual after distributing the discharge, which deviates from the original intention of distribution. Therefore, the initial flow should remain unchanged in this situation, which will not allow more loss of diversity of population. Likewise, something similar happens under the situation of *Q*__avg_<*Q*_*min*_.

#### Strategy 2: Penalty function processing

Finding a solution that satisfies all the DHTS problem constraints is quite difficult. The penalty function is deemed as one of the effective methods to handle constraints [48]. It tries to force the unconstrained optimum towards the feasibility boundary by incorporating penalty terms into the fitness function that violates the constraints. The system constraints in this numerical example include the discharge limits of hydro plants, storage capacity limits of hydro plants, hydropower generation limits and thermal generation limits. In order to facilitate calculation, each penalty item has been standardized. So the penalty function is founded as follow,

$$F=\underset{i\in R_{s}}{\sum }F_{i}(P_{s,i})+\omega ∙\left(\underset{i\in R_{s}}{\sum }\left|\zeta (P_{s,i})\right|+\underset{i\in R_{h}}{\sum }\left|\phi (V_{h,i})\right|+\underset{i\in R_{h}}{\sum }\left|\psi (P_{h,i})\right|+\underset{i\in R_{h}}{\sum }\left|\varphi (Q_{h,24})\right|\right)$$ (26)

$$\left\{ \begin{array}{l} \zeta (P_{s,i})=\frac{P_{s,i}-\bar{P_{s}}}{\bar{P_{s}}}\\ \phi (V_{h,i})=\frac{V_{h,i}-\bar{V_{h}}}{\bar{V_{h}}}\\ \psi (P_{h,i})=\frac{P_{h,i}-\bar{P_{h}}}{\bar{P_{h}}}\\ \varphi (Q_{h,24})=\frac{Q_{h,24}-\bar{Q_{h}}}{\bar{Q_{h}}} \end{array}\right.$$ (27)

Where *ω* is penalty coefficient. *ζ*(*P*_*s*,*i*_), *ϕ*(*V*_*h*_,_*t*_), *ψ*(*P*_*h*_,_*t*_) and *φ*(*Q*_*h*_,_24_) are penalty terms from thermal power generation, storage capacity, hydropower generation and terminal discharge respectively. $\bar{P_{s}}$, $\bar{V_{h}}$, $\bar{P_{h}}$ and $\bar{Q_{h}}$ are the corresponding constraints.

#### Strategy 3: Selection strategy processing

In the process of searching, it is the possible to appear a super nectar resource, whose fitness is significantly higher than the others. This situation will lead that the search zone gradually approaches to the super resource and result in uncompleted search and premature phenomenon. At this point, the selection strategy based on ranking is proposed in the reference [49]. In rank selection, each individual of individual is sorted according to their objective values. Selection probability is only related to ranking value of the individual rather than their objective values. Rank selection provides a simple and effective way of controlling selective pressure and it has better robustness, which is proved to be an efficient way for hedging the occurrence risk of super one. And the selection probability is defined as follow,

$$\left\{ \begin{array}{l} P_{k}=\frac{1}{n}+a(t)∙\frac{n+1-2k}{n∙(n+1)}\\ a(t)=0.2+\frac{3t}{4N} \end{array}\right.k=1,2\cdots n;t=1,2\cdots N$$ (28)

Where *P*_*k*_ is the selection possibility of the nectar resource, whose rank is *k* in all *n* nectar resources. And *a*(*t*) is self-adaptive parameter, which increases with the increase of iterative generation *t*. *N* is the maximum iterative generations.

### The specific steps of EABC algorithm for solving DHTS problems

Step 1: Total number of bees is initialized *N*, in which one half are employers and the others are onlookers. The number of nectar resources is initialized *N*/2. Maximum residence times in each nectar resource is initialized *limit*. Iteration is marked iter = 0, Maximum iteration is initialized *Max*_*Cycle*.

Step2: The bees randomly select *N* nectar resources in solution space. Calculate fitness values with Eq (11) combining strategy 2 and sort the N nectar resources in the order of large to small by fitness values. The first *N*/2 nectar resources are regarded as initial populations.Deal with the initial populations according to strategy 1 and record the initial sign the res_times(1) = 0.

Step 3: Each employer randomly search around its located netar resource according to the Eq (19). Calculate each fitness value, if it is better than the original source, update the position of the employed bees and res_times(*i*) = 0. Otherwise, res_times(*i*+1) = res_times(*i*)+1.

Step 4: Calculate selection probability according to the strategy 3, each onlooker select a nectar reource to follow by the selection probability, update nectar resource by the greedy strategy and update residene times(i).

Step 5: Determine whether res_times(*i*) is greater than *limit*. If it is, continues. Otherwise, switch to step 7.

Step 6: The *i*th employer gives up its located nectar resource and searches for a new nectar resource according to Eq (20).

Step 7: Record the current optimal solution and iterations iter = iter+1.

Step 8: Determine whether iterations is greater than *Max*_*Cycle*. If it is, termination. Otherwise, skip to step 4.

### Computational experiments and results

**Fig 3:**
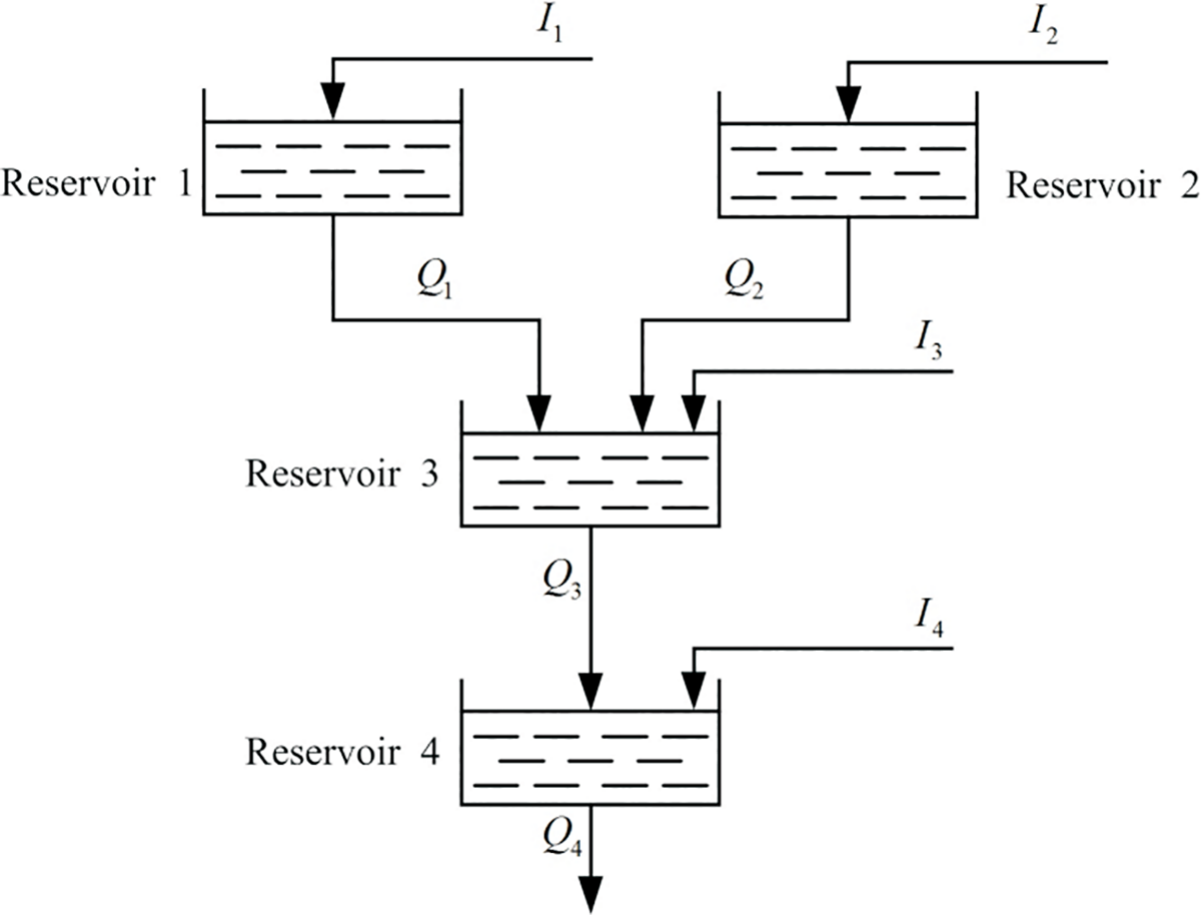
The network of hydraulic system.

In order to verify the effectiveness of the improved algorithm, a classic example of hydro-thermal economic scheduling system is introduced [25, 42–44].The daily hourly loads of the system are shown in Table 1 and the system contains four adjustable hydro plants and a thermal plant. The topological relationships of four hydro plants are shown in Fig 3 and the delay times between the hydro plants are shown in Table 2. The hydropower system limits are shown in Table 3. The hydropower generation coefficients and inflows of four reservoirs are shown in Tables 4 and 5, respectively. The thermal plant is simplified as a thermal power unit. The general cost coefficients of the thermal plant are 5000, 19.2 and 0.002, respectively. The composite minimum and maximum generations are 500 and 2500.

**Table 1:** The system daily hourly loads (MW).

Hour	1	2	3	4	5	6	7	8	9	10	11	12
Load	1370	1390	1360	1290	1290	1410	1650	2000	2240	2320	2230	2310
Hour	13	14	15	16	17	18	19	20	21	22	23	24
Load	2230	2200	2130	2070	2130	2140	2240	2280	2240	2120	1850	1590

**Table 2:** Time delay of the plant transform to direct downstream plant.

Plant	1	2	3	4
τ (h)	3	2	4	0

**Table 3:** Limits of the whole system.

Plant	*V*_*min*_	*V*_*max*_	*V*_*ini*_	*V*_*end*_	*Q*_*min*_	*Q*_*max*_	*Ph*_*min*_	*Ph*_*max*_
1	80	150	100	120	5	15	0	500
2	60	120	80	70	6	15	0	500
3	100	240	170	170	10	30	0	500
4	70	160	120	140	13	25	0	500

**Table 4:** Hydropower generation coefficients.

Plant	*C*_*1*_	*C*_*2*_	*C*_*3*_	*C*_*4*_	*C*_*5*_	*C*_*6*_
1	-0.0042	-0.42	0.030	0.90	10.0	-50
2	-0.0040	-0.30	0.015	1.14	9.5	-70
3	-0.0016	-0.30	0.014	0.55	5.5	-40
4	-0.0030	-0.31	0.027	1.44	14.0	-90

**Table 5:** Reservoir inflows.

Hour	Reservoir 1	Reservoir 2	Reservoir 3	Reservoir 4	Hour	Reservoir 1	Reservoir 2	Reservoir 3	Reservoir 4
1	10	8	8.1	2.8	13	11	8	4	0
2	9	8	8.2	2.4	14	12	9	3	0
3	8	9	4	1.6	15	11	9	3	0
4	7	9	2	0	16	10	8	2	0
5	6	8	3	0	17	9	7	2	0
6	7	7	4	0	18	8	6	2	0
7	8	6	3	0	19	7	7	1	0
8	9	7	2	0	20	6	8	1	0
9	10	8	1	0	21	7	9	2	0
10	11	9	1	0	22	8	9	2	0
11	12	9	1	0	23	9	8	1	0
12	10	8	2	0	24	10	8	0	0

The mathematical software of Matlab is used to simulate EABC algorithm for 25 times to calculate the numeral example, the maximal iterative generations *N*, the population size *n*, the *limit*, the guidance parameter *β* and crossover probability *P*_*c*_ are respectively set 2000, 40, 30, 1.5 and 0.9. The minimum cost of the 25 times is 922541, the corresponding solution and the hourly generations of each plant are given in Tables 6 and 7, which can match all constraints in each scheduling period.

**Table 6:** The best solution calculated from EABC algorithm.

Hour	hydro power generation(m^3^/s)			
	plant 1	plant 2	plant 3	plant 4
1	9.95	8.05	30.00	13.01
2	9.40	6.30	30.00	13.00
3	8.83	6.00	30.00	13.01
4	8.54	6.00	29.70	13.00
5	8.25	6.00	18.12	13.00
6	8.16	6.03	18.34	13.01
7	8.25	6.48	16.96	13.01
8	8.50	7.09	15.78	13.16
9	8.64	7.60	14.89	13.26
10	8.71	7.96	14.66	13.18
11	8.67	8.00	15.18	13.07
12	8.65	8.34	14.54	13.36
13	8.53	8.45	15.38	14.49
14	8.52	8.57	17.07	14.73
15	8.37	8.75	15.90	14.15
16	8.21	8.84	17.56	15.10
17	8.03	9.27	17.08	15.50
18	7.78	9.53	16.10	16.39
19	7.68	10.20	15.05	15.96
20	7.62	10.92	13.99	17.50
21	7.56	11.55	10.00	18.75
22	7.44	9.90	10.00	20.02
23	5.47	10.68	10.00	21.13
24	5.24	11.53	10.18	22.31

**Table 7:** Hourly generation of each plant.

Hour	hydro power generation(MW)				Thermal generation(MW)
	plant 1	plant2	plant 3	plant 4	
1	85.80	62.26	0.00	200.15	1021.79
2	82.97	52.04	0.00	187.75	1067.24
3	79.68	51.66	0.00	173.83	1054.84
4	77.48	53.28	0.00	156.76	1002.47
5	75.03	54.34	25.55	178.74	956.34
6	74.09	55.01	24.73	198.98	1057.19
7	74.52	57.97	29.98	217.45	1270.08
8	76.16	62.05	33.58	235.25	1592.96
9	77.42	65.56	35.77	240.75	1820.50
10	78.64	68.34	36.80	244.60	1891.62
11	79.46	69.12	36.42	246.87	1798.13
12	79.79	70.96	39.23	251.84	1868.18
13	79.79	71.37	39.73	263.15	1775.96
14	80.62	72.33	36.35	265.27	1745.43
15	80.29	73.45	41.17	260.78	1674.31
16	79.68	73.52	36.98	269.02	1610.81
17	78.66	74.61	39.27	272.50	1664.95
18	76.99	73.89	43.01	280.62	1665.49
19	76.16	75.19	46.21	276.94	1765.50
20	75.48	76.43	49.03	289.26	1789.80
21	74.95	77.19	50.70	296.49	1740.67
22	74.22	69.59	52.86	300.33	1623.00
23	59.01	71.20	54.65	299.60	1365.54
24	57.27	71.95	56.40	295.19	1109.19

**Fig 4:**
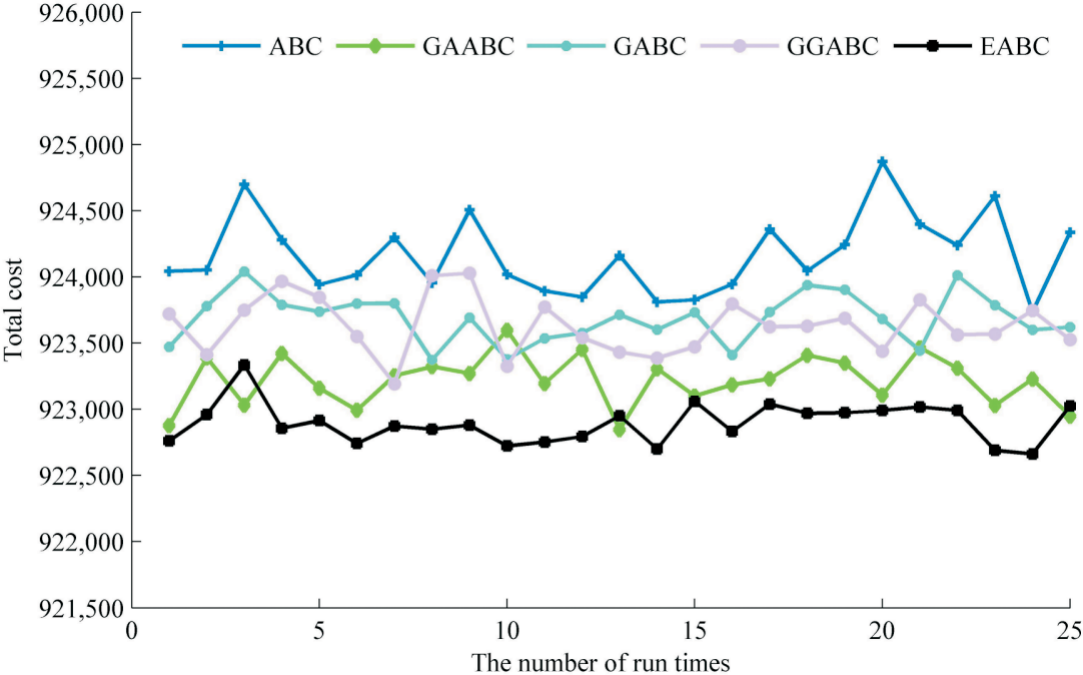
The best cost of each run time.

Similarly, we run ABC, GAABC (introduced only the idea of selection, crossover and mutation), GABC and GGABC (introduced only the gradient of global optimal solution) in turn with each for 25 times under the same conditions. The statistical results are given in Table 8 and distributions of each 25 optimal solutions are shown in Fig 4. In Table 8, all the minimum, average, maximum cost and standard deviation from GAABC by 25 tests are smaller than those of the standard ABC. It indicates that introducing the idea of selection, crossover and mutation for global search into ABC algorithm not only greatly improves the searching capability of the algorithm, but also makes the optimization effect more stable. Besides, compared the several parameters of GABC with that of GGABC, the introduced gradient information of global optimal solution still helps to enhance the searching ability even though the effect is less obvious than the former introduction. In Fig 4, we can easy find that all the 25 optimum solutions of the EABC are smaller than that of ABC and the modified effect is more stable. In summary, the two improvements can effectively improve the performance of the original algorithm. Among of them, the performance of ABC algorithm is improved significantly by introducing the idea of selection, crossover and mutation into it, which follows that the prior information is great importance to global search.

**Table 8:** Comparison of the results of ABC algorithms.

Method	Minimum cost($)	Average cost($)	Maximum cost($)	Std.
ABC	923736	924165	924870	295
GAABC	922846	923219	923597	193
GABC	923373	923686	924040	215
GGABC	923192	923632	924027	186
EABC	922541	922893	923334	152

**Fig 5:**
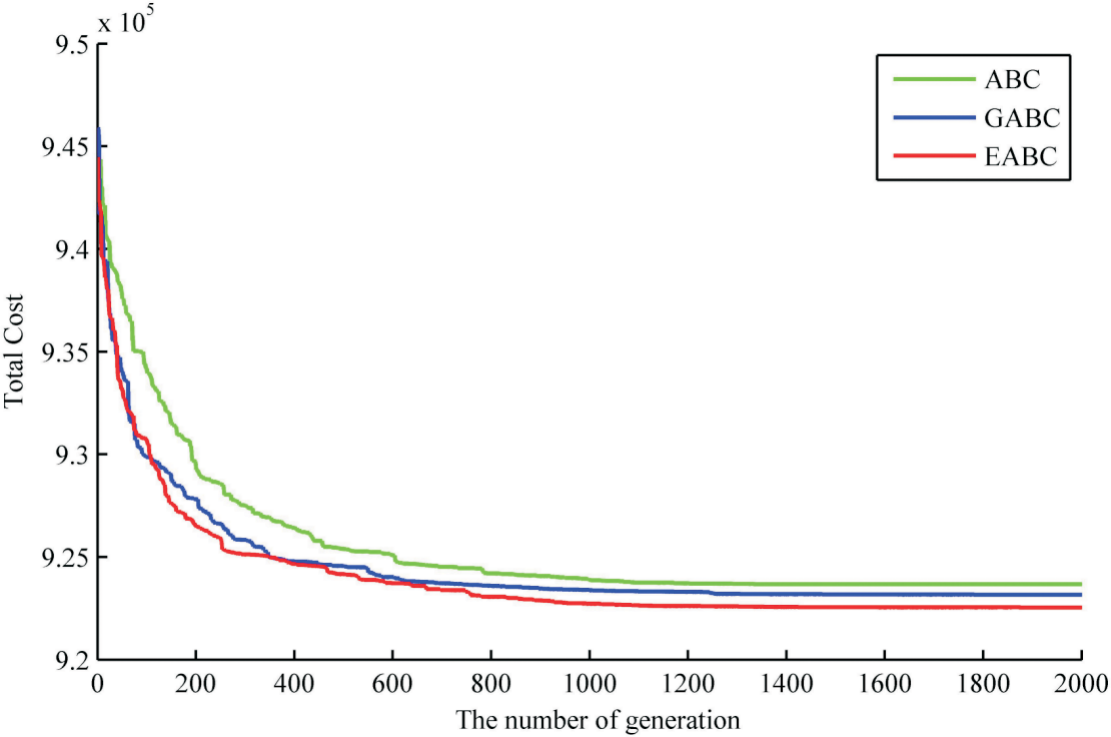
The optimization processes of the optimal solution with different algorithms.

Moreover, the optimization processes of the optimal solution with three selected algorithms are shown in Fig 5. It can be found that these algorithms have a higher optimization speed in the earlier stage, but drops it significantly in mid-late period. The optimization speed of GABC and EABC is higher than that of ABC in the process of optimization. In the first 100 generations, the optimization speed of EABC is almost the same with that of GABC, but more superior after 100 generations. In respect of optimization efficiency, GABC and EABC are also significantly better than ABC. EABC and GABC have similar optimal efficiency in almost the first 350 generations. After the 350 generations, the optimization effect of EABC is obviously better than GABC. From the above analysis, it is obvious that EABC has an excellent speed of convergence and optimization efficiency. Combined with the three algorithms’ 25 optimization results in Fig 5, which clearly shows that the performance of EABC is much superior than that of other algorithms.

In this paper, a numerical example is cited as a classic example, which has been calculated by GA[18], EGSA[25] and other algorithms[43, 50–55]. The optimization results of these literatures are listed in Table 9. In Table 9, compared with other algorithms,the minimum, average and maximum cost from EABC by 25 tests are all superior to others.It is evident that EABC has a better balance between exploration and exploitation.

**Table 9:** Comparison with other algorithms.

Method	Minimum cost($)	Average cost($)	Maximum cost($)
GA[18]	932734	936969	939734
CEP[25]	930166	930373	930927
FEP[25]	930268	930897	931397
PSO[25]	923418	924827	925938
EGSA[25]	922894	923223	923792
DE[25]	924751	925995	926742
IFEP[43]	930129	930290	930881
ACDE[50]	—	924661	—
MHDE[51]	—	925547	—
MAPSO[52]	—	924636	—
ORCCRO[53]	—	925195	—
MDE[54]	—	925960	—
RCGA[55]	930565	930966	931427
RCGA–AFSA[55]	927899	927963	928025
EABC	922541	922893	923335

This paper demonstrates separately the effectiveness of two proposed strategies in improving the calculation ability of ABC algorithm by comparing the results, and finally form an enhanced ABC algorithm which has excellent computing capacity. Compared with other algorithms, the improved ABC algorithm shows superiority in searching ability, which can provide decision support in quality for economic dispaching hydrothermal system.

## Conclusion

The DHTS problem is to utilize the cheap hydropower in priority, which takes advantage of reservoir regulation ability to adjust the output of hydro plant and then minimizes the cost of hydrothermal system. Various constraints should be considered over the scheduling period, including hydropower system constraints, thermal power system constraints, load demand and hydraulic connection etc. Obviously, the DHTS problem is essentially an optimization problem with high dimension and multiple nonlinear and nonconvex constraints. Considering the excellent performance of ABC algorithm in handling high dimension multi constraint of problems, the algorithm was introduced spontaneously to solve this kind of problems. This paper proposed an enhanced ABC algorithm to solve the DHTS problem, which used the global optimal solution and its gradient information to guide the local search algorithm to improve the exploitation, and selection, crossover and mutation operator to guide global search for scouter algorithms to improve the exploration. An excellent balance between exploitation and exploration of the algorithm was achieved finally. The results of this paper provide a new method for solving the DHTS problems and also provide a reference for the improvement and application of algorithms.

## Supporting information

S1 Table The system daily hourly loads (MW). (XLSX)

S2 Table Time delay of the plant transform to direct downstream plant. (XLSX)

S3 Table Limits of the whole system. (XLSX)

S4 Table Hydropower generation coefficients. (XLSX)

S5 Table Reservoir inflows. (XLSX)
